# Clinical *SMN1* and *SMN2* Gene-Specific Sequencing to Enhance the Clinical Sensitivity of Spinal Muscular Atrophy Diagnostic Testing

**DOI:** 10.1155/2023/6436853

**Published:** 2023-10-19

**Authors:** Cecelia R. Miller, Jin Fang, Pamela Snyder, Susan E. Long, Thomas W. Prior, Dan Jones, Matthew R. Avenarius

**Affiliations:** ^1^The Ohio State University Wexner Medical Center, Department of Pathology, 6100 Optometry Clinic & Health Science Faculty Office Building, 1664 Neil Avenue, Columbus, OH 43210, USA; ^2^The Ohio State University James Comprehensive Cancer Center Molecular Pathology Laboratory, 2001 Polaris Parkway, Columbus, OH 43240, USA; ^3^Case Western Reserve, Department of Pathology, 10900 Euclid Avenue, Cleveland, OH 44106, USA

## Abstract

**Purpose:**

Therapeutic advances in the treatment of spinal muscular atrophy (SMA) prompt the need for robust and efficient molecular diagnosis of this disease. Approximately five percent of SMA cases are attributable to one copy of *SMN1* with a hypomorphic or inactivating variant in *trans* with a deleted or converted allele. These intragenic variants are challenging to definitively localize to *SMN1* due to its sequence homology with the *SMN2* gene. To enhance the clinical sensitivity of SMA diagnostic testing, we present an optimized gene-specific sequencing assay to localize variants to either *SMN1* or *SMN2*.

**Methods:**

*SMN1* and *SMN2* genes are independently amplified by long-range allele-specific PCR. Long-range products are used in subsequent nested PCR reactions to amplify the coding exons of *SMN1* and *SMN2*. The resulting products are sequenced using standard Sanger-based methodologies and analyzed for disease-associated alterations.

**Results:**

83 probands suspicious for a clinical diagnosis of SMA with a nondiagnostic *SMN* dosage result were sequenced for intragenic variants in the *SMN1* gene. Gene-specific sequencing revealed likely disease-associated variants in *SMN1* in 42 cases (50.6%). Of the 42 variants, 27 are unique including 16 loss-of-function variants, 9 missense variants, 1 in-frame deletion variant, and 1 splice site variant.

**Conclusions:**

Herein, we describe an optimized assay for clinical sequencing of the full coding region of *SMN1* and *SMN2*. This assay uses standard techniques and equipment readily available to most molecular diagnostic laboratories.

## 1. Introduction

Spinal muscular atrophy (SMA) is an autosomal recessive motor neuron disease characterized by symmetric muscle atrophy and weakness secondary to anterior horn cell degeneration [1]. With an incidence of approximately 1 in 11,000 live births, SMA was considered the most common genetic cause of infant mortality; however, the recent approval of several therapies has drastically altered the natural progression of this disease [2]. Clinically, SMA disease presentations are heterogeneous with profound proximal muscle weakness, hypotonia, and early death observed in type I patients and decreasing severity with later presentations seen in types II, III, and IV [3].

SMA exhibits locus heterogeneity; however, the vast majority of SMA patients (~96%) segregate biallelic loss-of-function variants in the survival motor neuron 1 gene (*SMN1*), a gene that encodes a protein involved in small nuclear ribonucleoprotein biogenesis (snRNP) and pre-mRNA processing [4, 5]. Pathogenesis may relate to motor neuron dysfunction from abnormal snRNP assembly producing ineffectual splicing or abnormal mRNA trafficking [6]. The human genome encodes a paralogous gene, *SMN2*, that differs from *SMN1* by a single variant in the coding region (c.840C>T). While this synonymous variant does not impact the protein, it disrupts a critical exon splice enhancer site in *SMN2* that reduces its splicing efficiency by ~90% compared to *SMN1* [7, 8]. The residual full-length *SMN2* transcript partially rescues the cellular phenotype in SMA patients, with the severity of the disease reduced by each additional copy of *SMN2* present in the genome.

Recently, SMA management has moved beyond supportive care with the approval of several targeted therapies that demonstrate remarkable clinical outcomes [9, 10]. These “SMN-enhancing” strategies are directed at upregulating the *SMN2* full-length transcript using an antisense oligonucleotide approach or a gene replacement strategy that increases SMN protein levels above the threshold for disease. Regardless of treatment modality, clinical outcomes are best with early intervention underscoring the need for rapid molecular diagnostics. In 95% of cases, SMA results from homozygosity for *SMN1* gene deletion/conversion alleles, usually involving at least exon 8 (legacy nomenclature-exon 7) [11]. However, in ~5% of SMA cases, there is an intact copy of *SMN1* with a hypomorphic or inactivating allele in *trans* with a *SMN1* converted/deleted allele [12]. In these compound heterozygous cases, localizing the disease-associated variant to *SMN1* is confounded by the presence of the *SMN2* paralog requiring a different testing approach.

Previous gene-specific sequencing strategies in our laboratory for compound heterozygote localization have been incomplete (lacking coverage of exon 1) and in some instances gave inconsistent allele-specific amplification. These cases often required multiple sequencing attempts that could delay resulting and therapy initiation. Here, we report an optimized assay to localize variants to either *SMN1* or *SMN2*. This approach uses long-range allele-specific PCR to independently amplify the *SMN1* and *SMN2* loci followed by nested PCR and Sanger sequencing to amplify and sequence the coding regions of these genes. This approach allows for implementation of a gene-specific sequencing assay that is cost-effective and does not rely on sophisticated long read NGS sequencers or informatics that may be challenging for the routine molecular diagnostic laboratories to implement. Indeed, gene-specific localization of variants throughout the entire coding regions of the *SMN1* and *SMN2* genes allows for more comprehensive diagnostics thereby enhancing the ability to identify additional therapy-eligible patients.

## 2. Materials and Methods

### 2.1. Samples

*SMN1* and *SMN2* gene-specific sequencing was performed at The Ohio State University Molecular Pathology/James Molecular Laboratory on 9 probands from our own institution and 74 from other hospitals (Supplementary Table 1). The primary reason for referral was gene-specific sequencing for symptomatic patients harboring a single copy of *SMN1*, although symptomatic patients with alternate *SMN1* copy numbers were occasionally also sequenced. Genomic DNA was extracted from the referred specimens using the standard salting-out method or the EZ1 DNA Blood Kit (Qiagen, cat # 951054) [13].

### 2.2. SMN Copy Number Analysis

*SMN1* and *SMN2* copy number determination was carried out using a competitive dosage method. In brief, *SMN1* and *SMN2* were coamplified with an internal control and a calibrator. Resulting products were analyzed by capillary electrophoresis, and a semiquantitative approach was used for copy number determination as previously described [14].

### 2.3. *SMN* Gene Sequencing Strategy

First, the coding regions of *SMN1* (GRCh38/hg38; NM_000344.4) and *SMN2* (GRCh38/hg38; NM_017411.3) were coamplified and sequenced using standard Sanger-based methodologies (Supplementary Table 2). If a known pathogenic or uncharacterized variant was identified, the case was reflexed to gene-specific sequencing whereby *SMN1* and *SMN2* were independently amplified using allele-specific long-range PCR (Supplementary Figure 1). Subsequently, long-range products were assessed by nested PCR amplification of exon 8 and Sanger sequencing whereby the ratio of cytosine (C) from *SMN1* (GRCh38/hg38 chr 5 g. 70951946) to thymine (T) from *SMN2* (GRCh38/hg38 chr 5 g.70076526) was used to confirm *SMN1/SMN2* specificity. The exon harboring the putative disease-associated variant was also amplified and sequenced in a separate reaction to determine whether the variant originated from the *SMN1* or the *SMN2* long-range PCR product (Supplementary Methods).

## 3. Results

### 3.1. Redesign and Optimization of a SMN1 and SMN2 Gene-Specific Sanger Sequencing Assay

Given the lack of exon 1 coverage and inconsistent allele-specific amplification of our prior *SMN1/SMN2* sequencing assay, we designed a long-range PCR/sequencing assay that built upon the work of Kubo et al. [15]. Using a common *SMN1/SMN2* forward primer (SMN_FL_EX1-654_F) and the same exon 9 variant (*SMN1*–GRCh38/hg38 chr5 g.70952674; *SMN2–*GRCh38/hg38 chr5 g.70077254), a series of reverse primers were placed at varying positions and tested to identify the primer combination that yielded the highest degree of allele specificity (Supplementary Table 3). By comparing cytosine (*SMN1*) to thymine (*SMN2*) levels at coding position 840 (exon 8), the *SMN1*-specific reverse primer, SMN1_FL_EX8_R_16, and the *SMN2*-specific reverse primer, SMN2_FL_EX8_R_3, gave the highest degree of gene-specific amplification.

Using these optimized primer pairs, increased specificity was further achieved by introducing a dimethyl sulfoxide (DMSO) gradient with a concentration of 7% DMSO (range: 6%-15%) being optimal (Supplementary Figure 2). Finally, additional allele specificity was achieved by decreasing the molar concentration of the allele-specific reverse primer compared to the common forward primer (Supplementary Figure 3) [16].

### 3.2. Accuracy of the SMN1 and SMN2 Gene-Specific Sequencing Assay

**Figure 1:**
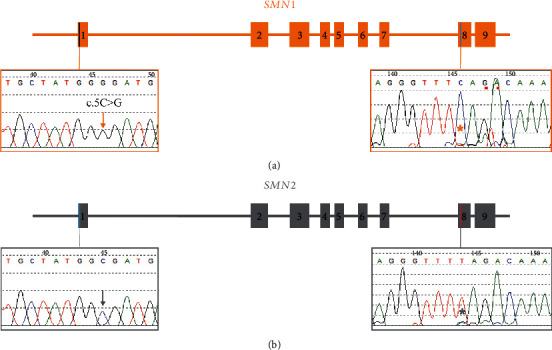
Gene-specific sequencing localizes the c.5C>G (p.Ala2Gly) variant to the *SMN1* gene. The coding exons of *SMN1* and *SMN2* were screened using previously validated *SMN1*/*SMN2* (non-gene-specific) primers to identify the c.5C>G (p.Ala2Gly) substitution in exon 1 of the *SMN1* or *SMN2* gene. Using the newly designed assay, long-range gene-specific PCR products were generated for *SMN1* (a) and *SMN2* (b). The long-range products were used as a template for nested PCR to amplify exons 1 and 8, and the resulting products were the Sanger sequenced. Differential amplification of *SMN1* and *SMN2* was demonstrated by a sole cytosine peak (orange asterisk) in the *SMN1*-derived product verse a sole thymine peak (gray asterisk) in the *SMN2*-derived product. At coding position 5, the *SMN2* product demonstrated the reference cytosine peak (gray arrow down) whereas the *SMN1* product demonstrated a nonreference guanine peak (orange arrow down), which is consistent with localization of the c.5C>G (p.Ala2Gly) variant to the *SMN1* gene.

To confirm accuracy of the new assay, 13 previously tested positive cases (with variants in each of the 8 coding exons of *SMN1* including five cases with exon 1 variants that could not be localized by the prior assay) and 12 negative cases were assessed. Using these optimized PCR conditions, all 25 cases showed gene-specific amplification as confirmed by C to T ratio at coding position 840 of exon 8 of each long-range product (Table 1). In the 8 positive cases with variants in exons 2-8, the *SMN1* versus *SMN2* localization of the sequence variant previously reported was confirmed. For the five exon 1 cases with the c.5C>G (p.Ala2Gly) variant, the new assay showed unequivocal *SMN1* localization (Figure 1). Additional studies to assess the reproducibility of this assay were conducted including intra-assay, inter-assay, and inter-technician studies, all of which demonstrated concordant results (data not shown). Although it was not observed, it is perceivable that residual genomic DNA in the long-range PCR reaction could affect the specificity of the assay.

### 3.3. Summary of Results in Our Entire Patient Cohort

Since *SMN1* and *SMN2* gene-specific sequence analysis was launched at our institution in 2005, a total of 83 probands have been analyzed, including 81 with our prior *SMN1/SMN2* sequencing assay. The median age at the time of referral was 3.72 years (range = 9 days to 76 years) and included 53 males, 27 females, and 3 cases where the sex was not reported. As expected, this cohort was enriched for cases with a single copy of *SMN1* (*n* = 65); however, 10 cases with two copies of *SMN1* and 8 cases where *SMN1* copy number was not assessed were also tested (Supplementary Table 1).

**Figure 2:**
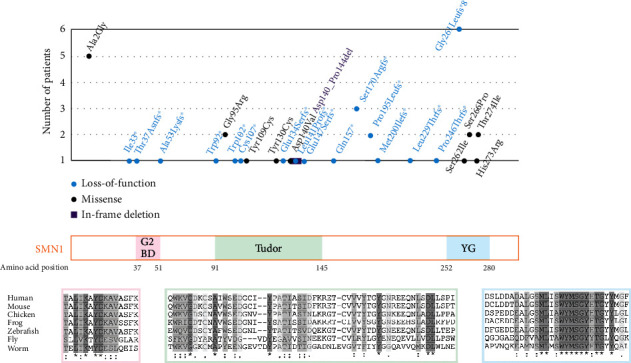
Summary of *SMN1* variants. The SMN1 protein is depicted above with multispecies amino acid alignments provided for each functional domain. The variants identified in this cohort are plotted relative to their position in the SMN1 protein, and their frequency is indicated on the *y*-axis. The *SMN1* splice site variant, c.835-3C>T, is not depicted above. G2BD (Gemin2-binding domain), Tudor (interaction with Sm proteins), and YG box (homodimerization).

After resolving the exon 1 cases with our new assay, forty-two cases harbored disease-associated variants in the *SMN1* gene (42/83; 50.6%) including 27 unique variants (16 premature stop variants, 9 missense variants, 1 in-frame deletion variant, and 1 splice site variant). The missense variants clustered in the Tudor domain (exon 4) and the YG box (exon 7), with the most frequently occurring missense variant being c.5C>G (p.Ala2Gly) in exon 1 (Figure 2 and Supplementary Table 1). Loss-of-function variants occurred throughout the *SMN1* gene with the most frequent being the c.770_780dup (p.Gly261LeufsTer8) variant in exon 7, which was seen in six cases (Figure 2 and Supplementary Table 1). The c.275G>A (p.Trp92^∗^) and c.684dupA (p.Leu229Thrfs^∗^27) variants, to our knowledge, have not been previously described in the literature and therefore represent novel findings. Noticeably absent from our cohort were the two most frequently reported variants in the literature, c.815A>G (p.Tyr272Cys) and c.399_402del (p.Arg133fsTer15) [17].

In forty of the remaining cases, a causative variant was not detected. Because this assay does not analyze the entirety of *SMN1* (i.e., noncoding regions), this result further reduces the possibility of a diagnosis of SMA. The final sample of our cohort tested positive for the c.223G>A (p.Ala75Thr) by an external laboratory; however, they were unable to determine the gene of origin, *SMN1* or *SMN2*. Using the methodology presented here, the c.223G>A (p.Ala75Thr) variant was localized to *SMN2*.

## 4. Discussion

We present the validation of an improved gene-specific sequencing assay for the detection of variants across the coding regions of *SMN1* and *SMN2*. Given advancements in SMA therapeutics, expanding molecular diagnostic testing for symptomatic individuals with a single *SMN1* allele is imperative for detecting therapy-eligible patients. Further, overcoming the technical hurdles associated with sequencing these paralogous genes contributes to more comprehensive testing for SMA.

The homology between *SMN1* and its paralog, *SMN2*, presents a significant challenge for independently sequencing these loci. While long-range allele-specific PCR methods have been used to successfully localize variants to the *SMN1* gene, these approaches have not necessarily offered highly reliable, full gene coverage. For example, one of the first sequencing assays failed to achieve coverage of exon 1 [18], which may have been due to the fidelity of the polymerase and its inability to accurately and efficiently copy across the entire *SMN1* and *SMN2* loci (~28.5 kB). More recently, an assay that achieved full gene coverage was described; however, in our experience, the specificity was variable [15]. It is important to note that the exact experimental parameters were not able to be replicated due to the unavailability of the polymerase. Due to these challenges (i.e., reagent reliability/availability, reproducibility, and limited coverage), the vast majority of clinical laboratories have elected not to perform gene-specific sequencing.

To achieve a high degree of specificity and reproducibility, long-range gene-specific amplification of the *SMN1* and *SMN2* loci required significant optimization including primer design, annealing temperature, DMSO concentration, and asymmetric primer concentrations. Numerous allele-specific primer pairs were designed to account for variables affecting allele specificity such as local sequence content and primer stability/thermodynamics. Additional measures including the presence of 7% DMSO and asymmetric primer concentration further contributed to achieving a high degree of gene-specific amplification. These variables, in addition to using commercially available products that have undergone rigorous quality control processes/certification, have contributed to the success of this gene-specific sequencing assay.

Our approach is advantageous because it uses established laboratory technologies that allow for expeditious and cost-efficient testing. Secondly, detection of variants by this DNA-based sequencing approach is not complicated by variants impacting transcript stability that have been seen with RNA-based approaches and it provides an opportunity to pursue family studies. Lastly, long-read sequencing technologies may also be used for sequencing the *SMN* paralogs, but the bioinformatic challenges associated with data processing along with the cost of implementation may prohibit routine diagnostic laboratories from adapting this technology, at least in the short term [19]. In the long term, this assay may be used as an orthogonal method of confirmation for long-range sequencing results and targeted variant detection.

While the optimized assay presented here overcomes the technical difficulties associated with sequencing the *SMN* paralogs, challenges remain in variant interpretation. The missense variants identified here cluster in exons 4 and 7 whereas loss-of-function variants localize throughout the coding region of *SMN1* (Figure 2), as has been noted in other studies [20]. Given the mechanism of disease, loss-of-function variants in symptomatic individual with a single copy of *SMN1* typically meet likely pathogenic criteria. Missense variants, on the other hand, remain challenging due to limited functional studies and a lack of *SMN1*- and *SMN2*-specific population-level sequence data. Albeit less common, missense variants may elicit splicing defects that result in human disease [21]. While this mechanism has not been associated with SMA, it cannot be discounted. In any case, novel *SMN1* missense variants could only achieve a classification of “variants of uncertain significance” using the existing guidelines [22].

There are some functional data that would support pathogenic classification in a subset of missense variants. In particular, missense variants localizing to the Tudor domain, a protein interaction domain that binds core spliceosomal (Sm) proteins, have the most obvious structural correlates. Disruption of the charge distribution within this domain, as is the case for the c.400G>A (p.Glu134Lys) variant, has been shown to abolish interaction with Sm proteins [23]. Supporting this case, we identified a symptomatic patient with a single copy of *SMN1* and an in-frame deletion, c.418_432del (p.Asp140_Pro144), that would produce a similar charge distribution shift in this domain. In this disease-specific context, we argue that such missense variants affecting evolutionarily conserved residues within well-characterized functional domains can be regarded as likely pathogenic variants in patients with a single copy of *SMN1*. It is especially prudent to factor clinical data into the assessment in these cases, with cases presenting with SMA phenotypes providing additional evidence for disease association.

Further, classification of disease-associated variants will require additional resources such as more extensive population-based *SMN1*- and *SMN2-*specific sequencing to understand the frequency of variants in various populations. Variant interpretation would also benefit from the development of *in vitro* screens to rigorously test the functional consequence of novel missense variants. Establishing “disease-specific” pathogenicity criteria will also require cross-laboratory collaborations given the relative rarity of missense variants in SMA.

Lastly, the utility of this assay may also be used to further rule out a diagnosis of SMA. In this context, an apparently asymptomatic newborn with a single copy of *SMN1* tested positive for a c.223G>A (p.Ala75Thr) variant by an external laboratory; however, the laboratory could not localize the variant to *SMN1* or *SMN2*. Using the assay described here, our laboratory definitively localized the c.223G>A (p.Ala75Thr) variant to *SMN2*. This result further illustrates the utility of this clinical assay in that the possibility of a SMA diagnosis was drastically reduced for this patient.

## 5. Conclusions

Given the need for a definitive SMA diagnosis prior to initiating costly therapeutic interventions, we present an optimized assay for the molecular diagnosis of SMA patients with compound heterozygous variants in the *SMN1* gene. This assay is cost-effective and may be adapted by nearly any clinical molecular diagnostic laboratory. While the methodology presented here overcomes the gene-specific localization of variants to *SMN1* or *SMN2*, unique challenges for interpreting sequence variants due to the lack of population data remains; however, recent long-read *SMN1* and *SMN2* locus-specific sequencing approaches may help overcome this gap. Working towards comprehensive detection and a better understanding of *SMN1* variants is essential for reliably diagnosing and identifying therapy-eligible patients.

## Figures and Tables

**Table 1:** Accuracy results for *SMN1* and *SMN2* gene-specific validation cohort.

Case ID	Copy number (*SMN1*, *SMN2*)	Exon	Gene	Previous clinical sequencing result	This study
1	1, 1	1	*SMN1*	c.5C>G	c.5C>G
			*SMN2*	c.5C>G	No variant detected
2	n/d	1	*SMN1*	c.5C>G	c.5C>G
			*SMN2*	c.5C>G	No variant detected
3	1, 1	1	*SMN1*	c.5C>G	c.5C>G
			*SMN2*	c.5C>G	No variant detected
4	1, 1	1	*SMN1*	c.5C>G	c.5C>G
			*SMN2*	c.5C>G	No variant detected
5	1, 2	1	*SMN1*	c.5C>G	c.5C>G
			*SMN2*	c.5C>G	No variant detected
6	1, 2	2	*SMN1*	c.93_96dupTGAC	c.93_96dupTGAC
			*SMN2*	No variant detected	No variant detected
8	1, 2	3	*SMN1*	c.156_165delinsCA	c.156_165indelCA
			*SMN2*	No variant detected	No variant detected
10	1, 2	4	*SMN1*	c.283G>C	c.283G>C
			*SMN2*	No variant detected	No variant detected
16	1, 2	4	*SMN1*	c.399_402delAGAG	c.399_402delAGAG
			*SMN2*	No variant detected	No variant detected
24	1, 3	5	*SMN1*	c.510_511delTG	c.510_511delTG
			*SMN2*	No variant detected	No variant detected
28	1, 2	6	*SMN1*	c.684dupA	c.684dupA
			*SMN2*	No variant detected	No variant detected
31	1, 1	7	*SMN1*	c.770_780dupCTGATGCTTTG	c.770_780dupCTGATGCTTTG
			*SMN2*	No variant detected	No variant detected
41	1, 3	7	*SMN1*	c.821C>T	c.821C>T
			*SMN2*	No variant detected	No variant detected

NM_000344.4 transcript was used to annotate *SMN1* variants. n/d: specimen was referred with no copy number data. Data not shown for negative cases (case ID: 84-95).

## Data Availability

The authors acknowledge that all data supporting the conclusions made by this study are available in the manuscript or as supplementary material.

## Supplementary Materials

Supplementary Materials Supplementary material includes three figures (experimental overview, DMSO optimization, and primer concentration optimization), one section dedicated to description of gene-specific methodology, and three supplementary tables (all gene-specific sequencing results and *SMN1* and *SMN2* copy number, SMN PCR primers, and *SMN1/SMN2* gene-specific PCR primers).
