# Supplementary material for: Clinical SMN1 and SMN2 Gene-Specific Sequencing to Enhance the Clinical Sensitivity of Spinal Muscular Atrophy Diagnostic Testing
Source: Hum Mutat. 2023 Oct 19;2023:6436853. doi: 10.1155/2023/6436853 (PMC11919053; doi:10.1155/2023/6436853)
Supplement: Supplementary Materials — Supplementary material includes three figures (experimental overview, DMSO optimization, and primer concentration optimization), one section dedicated to description of gene-specific methodology, and three supplementary tables (all gene-specific sequencing results and SMN1 and SMN2 copy number, SMN PCR primers, and SMN1/SMN2 gene-specific PCR primers). [file 6436853.f1.docx]

**Supplementary material:** Miller CR *et al*. Clinical *SMN1* and *SMN2* gene-specific sequencing for enhanced clinical sensitivity of spinal muscular atrophy diagnostic testing

**
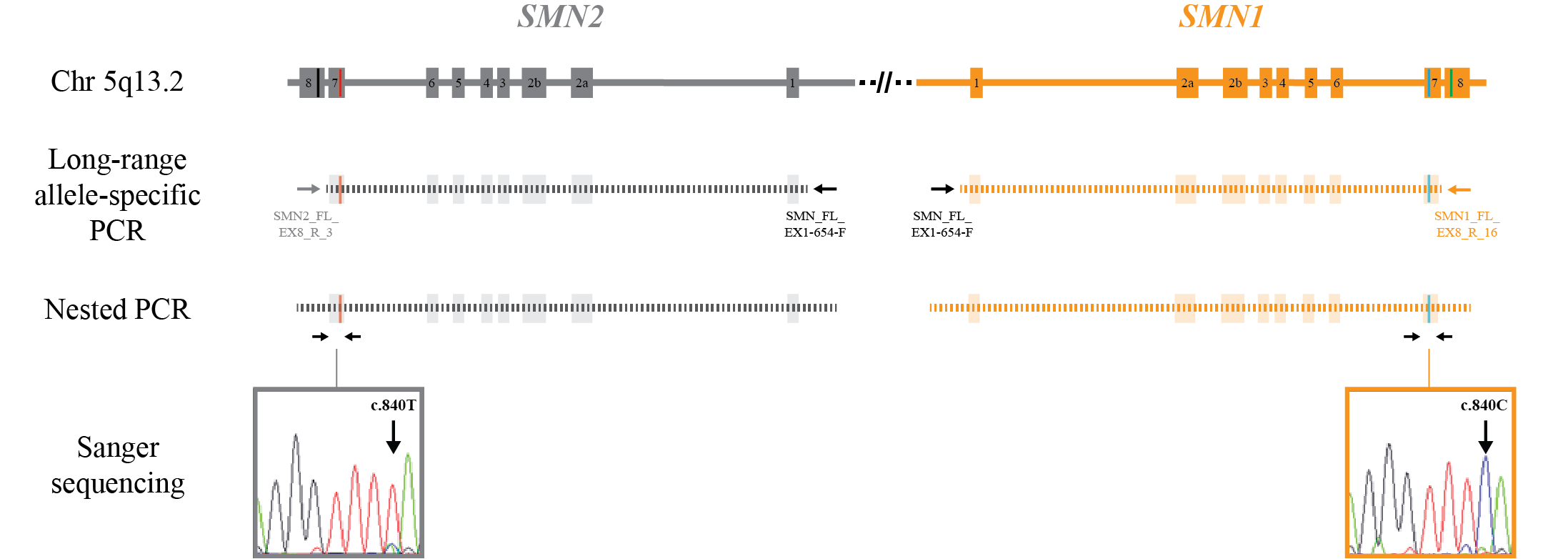
**

**Supplementary Figure 1.** **Experimental strategy for *SMN1* and *SMN2* gene-specific sequencing. Step 1 (not shown):** An initial screen is performed using common, non-gene-specific primers to simultaneously amplify and sequence the coding regions of *SMN1* and *SMN2*. **Step 2:** Independent long-range allele-specific PCR is performed with a common forward primer (SMN_FL_EX1-654-F) and allele-specific reverse primers (*SMN1:* SMN1_FL_EX8_R_16 & *SMN2:* SMN2_FL_EX8_R_3). **Step 3**: The resulting long-range products are subsequently used as template for nest-PCR reactions to amplify exon 7 and the exon harboring the variant identified in step 1. **Step 4**: Nested PCR products are Sanger sequenced and analyzed for gene-specificity (i.e., ratio of cytosine (*SMN1*)/thymine (*SMN2*) signal at coding position 840 of exon 7) and to determine whether the variant originated from the *SMN1* verses *SMN2* long-range product.

**
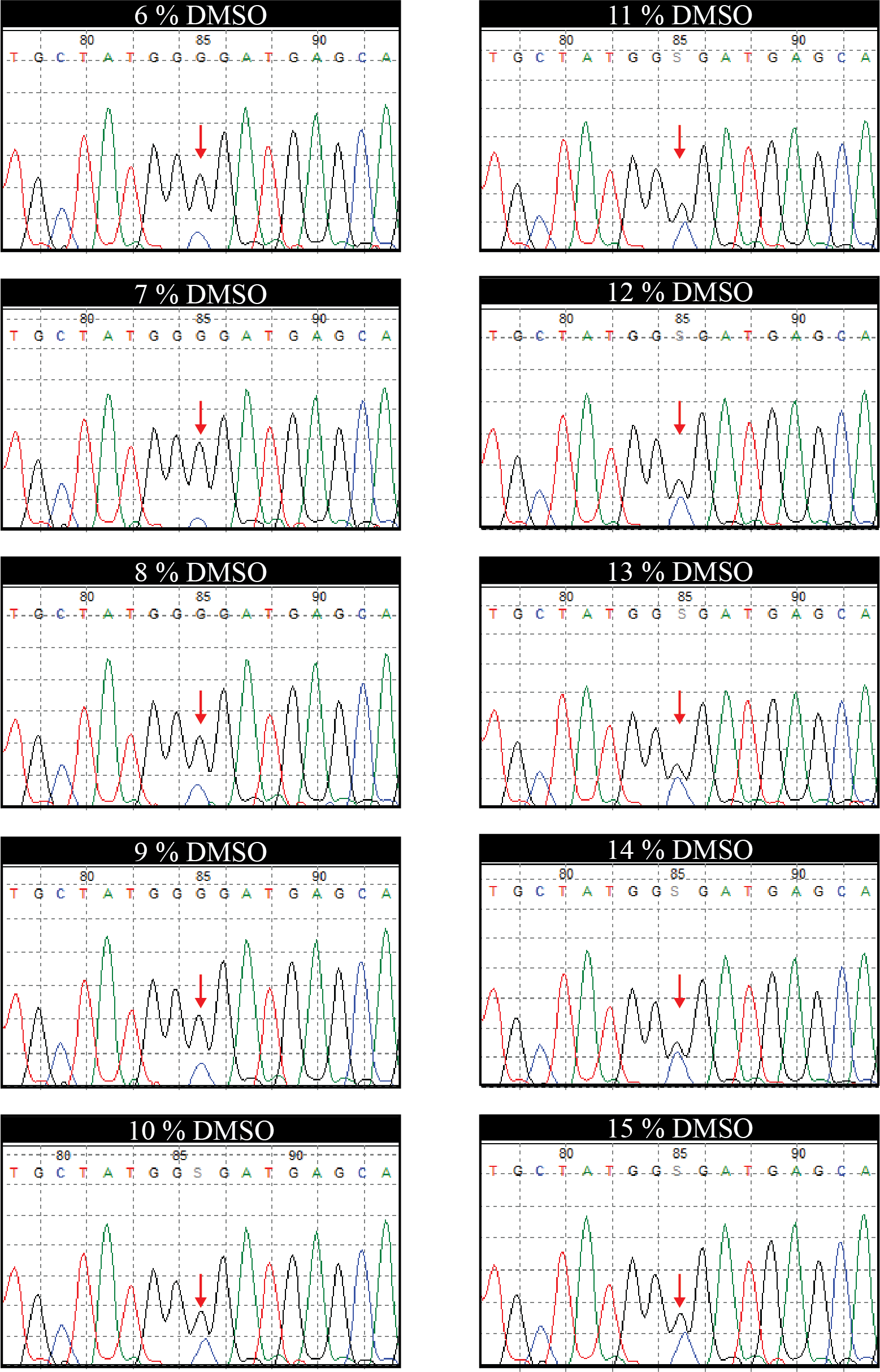
**

**Supplementary Figure 2. Long-range PCR optimization – DMSO gradient.** To ensure allele specificity throughout the entire locus, DNA from a case with the exon 1 c.5C>G (p.Ala5Gly) variant was used in a series of long-range gene-specific (*SMN1*) reactions with varying amounts of dimethyl sulfoxide (range = 6% - 15%). The optimal concentration was 7% dimethyl sulfoxide.

**
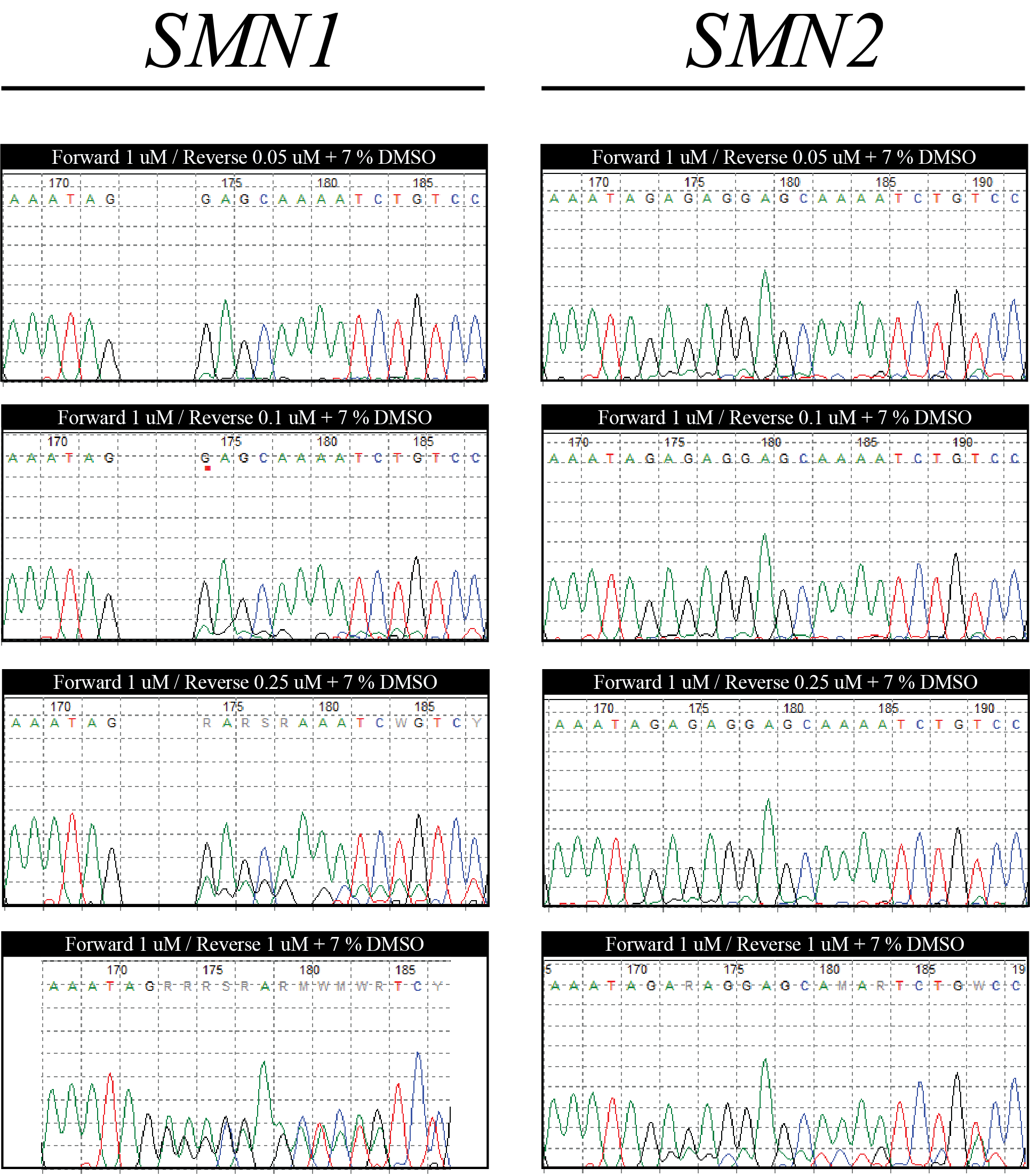
**

**Supplementary Figure 3. Long-range PCR optimization – Allele-specific primer gradient.** DNA from a case with an exon 3 variant, c.399_402del (p.Glu134Serfs*14), was used in a series of long-range gene-specific (*SMN1* & *SMN2*) reactions with 7% dimethyl sulfoxide and varying molar concentrations of the reverse (allele-specific) primer (range 1 µM – 0.05 µM). The optimal molar concentration is 0.05 µM for *SMN1* and 0.1 µM for *SMN2*. In this experimental series, the forward primer was held constant at 1 µM.

**Supplementary Methods - *SMN1* and *SMN2* gene-specific sequencing protocol**

***SMN1*-specific long-range PCR reaction:** A 20 μL reaction was assembled to amplify the *SMN1* locus including 4 μL genomic DNA (50 ng/ul), 5 μL 4xUltraRun LongRange PCR Master Mix (Qiagen, Hilden, Germany; cat no 206442), 2 μL 10 μm SMN_FL_EX1-654_F, 1 μL 1 μm SMN1_FL_EX8_R_16 (1 µM), 1.4 μL DMSO (Qiagen, Hilden, Germany), and 6.6 μL water.

| ***SMN1*-specific long-range PCR reaction** | |
| --- | --- |
| **PCR Component** | **1 x reaction** |
| Long Range PCR Master Mix (4x) | 5 µl |
| DMSO | 1.4 µl |
| H_2_O | 6.6 µl |
| SMN_FL_EX1-654_F (10 µM) | 2 µl |
| SMN1_FL_EX8_R_16 (1 µM) | 1 µl |
| DNA (50 ng/µl) | 4 µl |
| Total volume | 20 µl |

***SMN2*-specific long-range PCR reaction:** A 20 μL reaction was assembled to amplify the *SMN2* locus including 4 μL genomic DNA (50 ng/ul), 5 μL 4xUltraRun LongRange PCR Master Mix (Qiagen, Hilden, Germany; cat no 206442), 2 μL 10 μm SMN_FL_EX1-654_F, 2 μL 1 μm SMN1_FL_EX8_R_16 (1 µM), 1.4 μL DMSO (Qiagen, Hilden, Germany), and 5.6 μL water.

| ***SMN2*-specific long-range PCR reaction** | |
| --- | --- |
| **PCR Component** | **1 x reaction** |
| Long Range PCR Master Mix (4x) | 5 µl |
| DMSO | 1.4 µl |
| H_2_O | 5.6 µl |
| SMN_FL_EX1-654_F (10 µM) | 2 µl |
| SMN2_FL_EX8_R_3 (1 µM) | 2 µl |
| DNA (50 ng/µl) | 4 µl |
| Total volume | 20 µl |

***SMN1* and *SMN2* long-range PCR thermocycling conditions:** Initial denaturation at 93ᵒ for 3 minutes, 20 cycles of denaturation at 93ᵒ for 20 seconds and annealing at 65.5 for 15 minutes, with a final extension of 72ᵒ for 10 minutes. All steps of the long-range PCR programs were carried out at a maximum ramp rate of 5ᵒ/sec to best assure the polymerase stays associated.

| ***SMN1* and *SMN2* gene-specific PCR cycling conditions** | | |
| --- | --- | --- |
| **Temperature** | **Time** | **Cycles** |
| 93 °C | 3 minutes | 1x |
| 93 °C | 20 seconds | 20x |
| 65.5 °C * | 15 minutes |  |
| 72 °C | 10 minutes | 1x |
| 12 °C | hold |  |
| * max ramp speed of 5 °C | |  |

**Nested PCR reaction:** Long-range PCR products are used in a series of nested PCR reactions to amplify the exon 7 of *SMN1* and *SMN2* (to assess specificity) and the exon harboring the variant identified by *SMN* gene sequencing. Using 1 μL of the long-range PCR product as a template, the reaction included 12.5 μL 2x Qiagen HotStar Mastermix (Qiagen, Hilden, Germany), 0.5 μL of exon-specific forward and reverse primer (5 μM), 1.5 μL 25mM MgCl_2_ and 9 μL water.

| **Nested PCR reaction** | |
| --- | --- |
| **PCR Component** | **1 x reaction** |
| HotStar Taq Master Mix | 12.5 µl |
| MgCl_2_ (25 mM) | 1.5 µl |
| Primer F (5 µM) | 0.5 µl |
| Primer R (5 µM) | 0.5 µl |
| H_2_O | 9 µl |
| Long-range PCR product | 1 µl |
| Total volume | 25 µl |

**Nested PCR thermocycling conditions:**  The nested PCR reactions were cycled as follows: initial denaturation of 95ᵒ for 15 minutes, followed by 30 cycles of denaturation at 95° for 30 seconds, annealing at 57° for 30 seconds, with an extension at 72° for 30 seconds. The cycling conditions concluded with a final extension of 72° for 8 minutes.

| **PCR cycling conditions** | | |
| --- | --- | --- |
| **Temperature** | **Time** | **Cycles** |
| 95 °C | 15 minutes | 1x |
| 95 °C | 30 seconds | 30x |
| 57 °C | 30 seconds |  |
| 72 °C | 30 seconds |  |
| 72 °C | 8 minutes | 1x |
| 4 °C | hold |  |

**Sanger sequencing:** The resulting nested PCR products were purified using the ExoSAP-IT Express reagent (ThermoFisher Scientific, Waltham, MA) and cycle sequencing performed using the ABI BigDye Terminator v3.1 Cycle Sequencing kit (Applied Biosystems, Foster City, CA). Following the sequence reaction, the samples were purified using the BigDye Xterminator kit (Applied Biosystems, Foster City, CA) and analyzed on the ABI 3730XL Genetic Analyzer (Applied Biosystems, Foster City, CA).

**Supplementary Table 1. Summary of *SMN1* and *SMN2* variants detected in patient cohort referred for gene-specific sequencing.**

The NM_000344.4 transcript was used to annotate SMN1 variants and the NM_017411.3 transcript was used to annotate SMN2 variants.

na = not applicable; n/d = no data; bold text = novel variant/not previously reported in the literature.

|  | ***SMN1* variant** | **Protein** | **Location** | ***SMN1* copy number** | ***SMN2* copy number** |
| --- | --- | --- | --- | --- | --- |
| 1 | c.5C>G | p.Ala2Gly | Exon 1 | 1 | 1 |
| 2 | c.5C>G | p.Ala2Gly | Exon 1 | n/d | n/d |
| 3 | c.5C>G | p.Ala2Gly | Exon 1 | 1 | 1 |
| 4 | c.5C>G | p.Ala2Gly | Exon 1 | 1 | 2 |
| 5 | c.5C>G | p.Ala2Gly | Exon 1 | 1 | 1 |
| 6 | c.93_96dup | p.Ile33* | Exon 2 | 1 | 2 |
| 7 | c.109dupA | p.Thr37Asnfs*8 | Exon 2 | 1 | 2 |
| 8 | c.156_165delinsCA | p.Ala53Lysfs*3 | Exon 3 | 1 | 1 |
| **9** | **c.275G>A** | **p.Trp92*** | **Exon 4** | **1** | **2** |
| 10 | c.283G>C | p.Gly95Arg | Exon 4 | 1 | 2 |
| 11 | c.283G>C | p.Gly95Arg | Exon 4 | 1 | 1 |
| 12 | c.305G>A | p.Trp102* | Exon 4 | 1 | 3 |
| 13 | c.321C>A | p.Cys107* | Exon 4 | n/d | n/d |
| 14 | c.326A>G | P.Tyr109Cys | Exon 4 | 1 | 2 |
| 15 | c.389A>G | p.Tyr130Cys | Exon 4 | 1 | 2 |
| 16 | c.399_402del | p.Glu134Serfs*14 | Exon 4 | 1 | 2 |
| 17 | c.418_432delGATCTACTTTCCCCA | p.Asp140_Pro144del | Exon 4 | 1 | 3 |
| 18 | c.419A>T | p.Asp140Val | Exon 4 | n/d | n/d |
| 19 | c.422_428delTACTTTC | p.Leu141Profs*6 | Exon 4 | n/d | n/d |
| 20 | c.439_443delGAAGT | p.Glu147Serfs*2 | Exon 4 | 1 | n/d |
| 21 | c.469C>T | p.Gln157* | Exon 4 | 1 | 2 |
| 22 | c.510_511delTG | p.Ser170Argfs*9 | Exon 5 | 1 | n/d |
| 23 | c.510_511delTG | p.Ser170Argfs*9 | Exon 5 | 1 | 2 |
| 24 | c.510_511delTG | p.Ser170Argfs*9 | Exon 5 | 1 | 3 |
| 25 | c.584delC | p.Pro195Leufs*18 | Exon 5 | 1 | n/d |
| 26 | c.584delC | p.Pro195Leufs*18 | Exon 5 | 1 | 3 |
| 27 | c.599dupT | p.Met200Ilefs*56 | Exon 5 | 1 | 3 |
| **28** | **c.684dupA** | **p.Leu229Thrfs*27** | **Exon 6** | **1** | **2** |
| 29 | c.734dupC | p.Pro246Thrfs*10 | Exon 7 | 1 | 3 |
| 30 | c.770_780dupCTGATGCTTTG | p.Gly261Leufs*8 | Exon 7 | 1 | 2 |
| 31 | c.770_780dupCTGATGCTTTG | p.Gly261Leufs*8 | Exon 7 | 1 | 1 |
| 32 | c.770_780dupCTGATGCTTTG | p.Gly261Leufs*8 | Exon 7 | 1 | 3 |
| 33 | c.770_780dupCTGATGCTTTG | p.Gly261Leufs*8 | Exon 7 | 1 | 2 |
| 34 | c.770_780dupCTGATGCTTTG | p.Gly261Leufs*8 | Exon 7 | 1 | 2 |
| 35 | c.770_780dupCTGATGCTTTG | p.Gly261Leufs*8 | Exon 7 | 1 | 2 |
| 36 | c.785G>T | p.Ser262Ile | Exon 7 | 1 | 1 |
| 37 | c.796T>C | p.Ser266Pro | Exon 7 | 1 | 3 |
| 38 | c.796T>C | p.Ser266Pro | Exon 7 | 1 | 2 |
| 39 | c.818A>G | p.His273Arg | Exon 7 | n/d | n/d |
| 40 | c.821C>T | p.Thr274Ile | Exon 7 | n/d | n/d |
| 41 | c.821C>T | p.Thr274Ile | Exon 7 | 1 | 3 |
| 42 | c.835-3C>T | na | Intron 7 | 1 | n/d |
| 43 | negative | na | na | 1 | 3 |
| 44 | negative | na | na | 2 | 2 |
| 45 | negative | na | na | 1 | 1 |
| 46 | negative | na | na | 1 | n/d |
| 47 | negative | na | na | 2 | 2 |
| 48 | negative | na | na | 2 | 1 |
| 49 | negative | na | na | 1 | 1 |
| 50 | negative | na | na | 1 | 1 |
| 51 | negative | na | na | 1 | 3 |
| 52 | negative | na | na | 2 | 1 |
| 53 | negative | na | na | n/d | n/d |
| 54 | negative | na | na | 2 | 1 |
| 55 | negative | na | na | 1 | 2 |
| 56 | negative | na | na | 2 | 2 |
| 57 | negative | na | na | 1 | n/d |
| 58 | negative | na | na | 1 | 4 |
| 59 | negative | na | na | 2 | 2 |
| 60 | negative | na | na | 1 | 2 |
| 61 | negative | na | na | 1 | 3 |
| 62 | negative | na | na | n/d | n/d |
| 63 | negative | na | na | 1 | 0 |
| 64 | negative | na | na | 2 | 0 |
| 65 | negative | na | na | 1 | 3 |
| 66 | negative | na | na | 1 | 3 |
| 67 | negative | na | na | 1 | 3 |
| 68 | negative | na | na | 1 | 0 |
| 69 | negative | na | na | 2 | 2 |
| 70 | negative | na | na | 1 | 2 |
| 71 | negative | na | na | 1 | 2 |
| 72 | negative | na | na | 1 | 1 |
| 73 | negative | na | na | 1 | 1 |
| 74 | negative | na | na | 1 | 2 |
| 75 | negative | na | na | 1 | 1 |
| 76 | negative | na | na | 1 | 2 |
| 77 | negative | na | na | 1 | 3 |
| 78 | negative | na | na | 1 | 3 |
| 79 | negative | na | na | 1 | 3 |
| 80 | negative | na | na | 1 | 2 |
| 81 | negative | na | na | 1 | 2 |
| 82 | negative | na | na | 2 | 2 |
|  | ***SMN2* variant** | **Protein** | **Location** | ***SMN1* copy number** | ***SMN2* copy number** |
| 83 | c.223G>A | p.Ala75Thr | Exon 3 | 1 | 4 |

**Supplementary Table 2.** ***SMN1/SMN2* PCR primers**

| **Primer** | **Sequence** |
| --- | --- |
| Exon 1 F | F: 5' - tgtaaaacgacggccagtggcggaagtcgtcactctt - 3' |
| Exon 1 R | R: 5' - caggaaacagctatgaccgggtgctgagagcgctaata - 3' |
| Exon 2a F | F: 5' - tgtaaaacgacggccagtctgattaaacctatctgaacatg - 3' |
| Exon 2a R | R: 5' - caggaaacagctatgacccgtatgttatcaattcctttcca - 3' |
| Exon 2b F | F: 5' - tgtaaaacgacggccagtctgtgcaccaccctgtaacatg - 3' |
| Exon 2b R | R: 5' - caggaaacagctatgaccaaggactaatgagacatcc - 3' |
| Exon 3 F | F: 5' - tgtaaaacgacggccagtcgagatgatagtttgccctc - 3' |
| Exon 3 R | R: 5' - caggaaacagctatgaccctcatctagtctctgcttcc - 3' |
| Exon 4 F | F: 5' - tgtaaaacgacggccagtcacccttataacaaaaacctgc - 3' |
| Exon 4 R | R: 5' - caggaaacagctatgaccgagaggttaaatgtcccga - 3' |
| Exon 5 F | F: 5' - tgtaaaacgacggccagttgagtctgtttgacttcagg - 3' |
| Exon 5 R | R: 5' - caggaaacagctatgacctatcaaattgtatgtgaaagca - 3' |
| Exon 6 F | F: 5' - tgtaaaacgacggccagtgcaaaaatacaattaatttccagc - 3' |
| Exon 6 R | R: 5' - caggaaacagctatgacctgcaagagtaatttaagcctcaga - 3' |
| Exon 7 F | F: 5' - tgtaaaacgacggccagtagactatcaacttaatttctgatc - 3' |
| Exon 7 R | R: 5' - caggaaacagctatgaccgtaagattcactttcataatgctg - 3' |

**Supplementary Table 3. Long-range gene-specific PCR primers**

| **Primer** | **Sequence** |  |  |
| --- | --- | --- | --- |
| SMN_FL_EX1-654_F | 5' - GTTGGGGGATCAAATATCTTCTAGTGTT - 3' | | |
| SMN2_FL_EX8_R_1 | 5' - CCCCCACC**T**CAGTCTTTTACAGATGGT - 3' | | |
| SMN2_FL_EX8_R_2 | 5' - CACCCCCACC**T**CAGTCTTTTACAGATG - 3' | | |
| SMN2_FL_EX8_R_3 | 5' - CCCACCCCCACC**T**CAGTCTTTTACAGA - 3' | | |
| SMN2_FL_EX8_R_4 | 5' - CTCCCACCCCCACC**T**CAGTCTTTTACA - 3' | | |
| SMN2_FL_EX8_R_5 | 5' - GCCTCCCACCCCCACC**T**CAGTCTTTTA - 3' | | |
| SMN2_FL_EX8_R_6 | 5' - TGGCCTCCCACCCCCACC**T**CAGTCTTT - 3' | | |
| SMN2_FL_EX8_R_7 | 5' - GCTGGCCTCCCACCCCCACC**T**CAGTCT - 3' | | |
| SMN2_FL_EX8_R_8 | 5' - GTGCTGGCCTCCCACCCCCACC**T**CAGT - 3' | | |
| SMN2_FL_EX8_R_9 | 5' - CCGTGCTGGCCTCCCACCCCCACC**T**CA - 3' | | |
| SMN2_FL_EX8_R_10 | 5' - ACCGTGCTGGCCTCCCACCCCCACC**T**C - 3' | | |
| SMN2_FL_EX8_R_11 | 5' - CACCGTGCTGGCCTCCCACCCCCACC**T** - 3' | | |
| SMN2_FL_EX8_R_36 | 5' - CCCACC**T**CAGTCTTTTACAGATGGTTT - 3' | | |
| SMN2_FL_EX8_R_37 | 5' - CACC**T**CAGTCTTTTACAGATGGTTTTT - 3' | | |
| SMN2_FL_EX8_R_38 | 5' - CC**T**CAGTCTTTTACAGATGGTTTTTCA - 3' | | |
| SMN2_FL_EX8_R_39 | 5' - **T**CAGTCTTTTACAGATGGTTTTTCAAA - 3' | | |
| SMN1_FL_EX8_R_1 | 5' - CACCGTGCTGGCCTGGCACCCCCACC**C** - 3' | | |
| SMN1_FL_EX8_R_2 | 5' - ACCGTGCTGGCCTGGCACCCCCACC**C**C - 3' | | |
| SMN1_FL_EX8_R_3 | 5' - CCGTGCTGGCCTGGCACCCCCACC**C**CA - 3' | | |
| SMN1_FL_EX8_R_4 | 5' - GTGCTGGCCTGGCACCCCCACC**C**CAGT - 3' | | |
| SMN1_FL_EX8_R_5 | 5' - GCTGGCCTGGCACCCCCACC**C**CAGTCT - 3' | | |
| SMN1_FL_EX8_R_6 | 5' - TGGCCTGGCACCCCCACC**C**CAGTCTTT - 3' | | |
| SMN1_FL_EX8_R_7 | 5' - GCCTGGCACCCCCACC**C**CAGTCTTTTA - 3' | | |
| SMN1_FL_EX8_R_8 | 5' - CTGGCACCCCCACC**C**CAGTCTTTTACA - 3' | | |
| SMN1_FL_EX8_R_9 | 5' - GGCACCCCCACCCCAGTCTTTTACAGA - 3' | | |
| SMN1_FL_EX8_R_10 | 5' - CACCCCCACC**C**CAGTCTTTTACAGATG - 3' | | |
| SMN_FL_EX8 | 5' - CCCCCACC**C**CAGTCTTTTACAGATGGT - 3' | | |
| SMN1_FL_EX8_R_11 | 5' - CCCACC**C**CAGTCTTTTACAGATGGTTT - 3' | | |
| SMN1_FL_EX8_R_12 | 5' - CACC**C**CAGTCTTTTACAGATGGTTTTT - 3' | | |
| SMN1_FL_EX8_R_13 | 5' - CC**C**CAGTCTTTTACAGATGGTTTTTCA - 3' | | |
| SMN1_FL_EX8_R_14 | 5' - C**C**CAGTCTTTTACAGATGGTTTTTCAA - 3' | | |
| SMN1_FL_EX8_R_15 | 5' - **C**CAGTCTTTTACAGATGGTTTTTCAAA - 3' | | |
| **SMN1_FL_EX8_R_16** | **5' - CCCACCCCCACCCCAGTCTTTTACAGA - 3'** | | |
| SMN1_FL_EX8_R_17 | 5' - CACCCCCACC**C**CAGTCTTTTACAGA - 3' | | |
